# Ilexsaponin A_1_ Ameliorates Diet-Induced Nonalcoholic Fatty Liver Disease by Regulating Bile Acid Metabolism in Mice

**DOI:** 10.3389/fphar.2021.771976

**Published:** 2021-12-14

**Authors:** Wen-wen Zhao, Meng Xiao, Xia Wu, Xiu-wei Li, Xiao-xi Li, Ting Zhao, Lan Yu, Xiao-qing Chen

**Affiliations:** ^1^ School of Traditional Chinese Medicine, Capital Medical University, Beijing, China; ^2^ Department of Pharmacy, Beijing Children’s Hospital, Capital Medical University, National Center for Children Health, Beijing, China; ^3^ National Institutes for Food and Drug Control, Beijing, China

**Keywords:** ilexsaponin A_1_, nonalcoholic fatty liver disease, bile acid, gut microbiota, farnesoid X receptor

## Abstract

Bile acid (BA) metabolism is an attractive therapeutic target in nonalcoholic fatty liver disease (NAFLD). We aimed to investigate the effect of ilexsaponin A_1_ (IsA), a major bioactive ingredient of *Ilex*, on high-fat diet (HFD)-induced NAFLD in mice with a focus on BA homeostasis. Male C57BL/6J mice were fed an HFD to induce NAFLD and were treated with IsA (120 mg/kg) for 8 weeks. The results showed that administration of IsA significantly decreased serum total cholesterol (TC), attenuated liver steatosis, and decreased total hepatic BA levels in HFD-induced NAFLD mice. IsA-treated mice showed increased BA synthesis in the alternative pathway by upregulating the gene expression levels of sterol 27-hydroxylase (CYP27A1) and cholesterol 7b-hydroxylase (CYP7B1). IsA treatment accelerated efflux and decreased uptake of BA in liver by increasing hepatic farnesoid X receptor (FXR) and bile salt export pump (BSEP) expression, and reducing Na^+^-taurocholic acid cotransporting polypeptide (NTCP) expression. Alterations in the gut microbiota and increased bile salt hydrolase (BSH) activity might be related to enhanced fecal BA excretion in IsA-treated mice. This study demonstrates that consumption of IsA may prevent HFD-induced NAFLD and exert cholesterol-lowering effects, possibly by regulating the gut microbiota and BA metabolism.

## Introduction

Nonalcoholic fatty liver disease (NAFLD), recently named metabolic-associated fatty liver disease (MAFLD), is a rapidly growing metabolic disease associated with type-2 diabetes and obesity (Tilg et al., 2021). NAFLD is a leading etiology of chronic liver disease affecting over 25% of the global population, which demands an exploration of robust treatment methods urgently (Younossi et al., 2020). Bile acid (BA) synthesis and excretion are critical in the progression of NAFLD, and also represent major pathways of cholesterol catabolism (Sciarrillo et al., 2021). Many drugs targeting BA metabolism have been shown to be effective against metabolic diseases, including apical sodium-dependent bile acid transporter (ASBT) inhibitors and FXR agonists/antagonists (Matye et al., 2021a; Matye et al., 2021b; Clifford et al., 2021). Studies highlighted efforts to activate BA synthesis, accelerate BA circulation, and fecal excretion as potential therapeutic strategies for NAFLD (Jia et al., 2020). Farnesoid X receptor (FXR) is a BA-activated nuclear receptor that modulates the transcriptional regulation of many targets, including genes encoding hepatic BA-metabolizing enzymes and BA/organic anion transporters. BA synthesis is regulated by several enzymes, e.g., cholesterol 7α-hydroxylase (CYP7A1) and sterol 12α-hydroxylase (CYP8B1) in the classical pathway, and cholesterol 7b-hydroxylase (CYP7B1) and sterol 27-hydroxylase (CYP27A1) in the alternative pathway (Hylemon et al., 2009). The activities of these enzymes are negatively regulated by the ileal FXR/fibroblast growth factor 15 (FGF15) axis. Hepatic FXR activation regulates BA levels in the liver by inducing canalicular bile salt export pump (BSEP) expression to promote BA efflux and repressing Na^+^/taurocholate cotransporting polypeptide (NTCP) to reduce BA uptake (Appelman et al., 2021). BA metabolism and excretion are closely related to the gut microbiota. Bacteria with high bile salt hydrolase (BSH) activity promote BA deconjugation, and deconjugated BAs are hydrophobic and easily excreted into feces (Sayin et al., 2013; Geng and Lin, 2016).

Currently available treatments with demonstrated benefits against NAFLD include lipid-lowering agents (statins), insulin-sensitizing agents (e.g., pioglitazone), apical sodium-dependent bile acid transporter (ASBT) inhibitors, and antioxidants (e.g., vitamin E) (Cusi et al., 2016; Ge et al., 2019; Al-Baiaty et al., 2021; Lee et al., 2021). None of these treatments have been approved by the US Food and Drug Administration due to limited effectiveness and adverse effects (Rao et al., 2020; Pafili and Roden, 2021; Boeckmans et al., 2019; Caparros-Martin et al., 2017). Active natural products have been paid much attention because of safe chronic application and their capacity to alleviate metabolic diseases (Ahmed et al., 2019; Xu et al., 2020).

The leaves of *Ilex* are commonly used in folk medicine for treating hypertension and dyslipidemia. We found that the triterpenoid saponin extract from *I. hainanensis* could alleviate liver inflammation in NAFLD mice (Zhao et al., 2019). Ilexsaponin A_1_ (IsA) is the most abundant triterpenoid saponin in *Ilex*, with demonstrated antithrombotic and anticoagulant effects (Zhou et al., 2011). What is more, we found that this saponin extract could reduce serum cholesterol and increase the relative abundance of bacteria associated with BA metabolism such as *Bacteroides*. In this study, we selected the main component, IsA, to investigate its effects on high-fat diet (HFD)-induced NAFLD, and further explored its role in BA homeostasis.

## Materials and Methods

### Preparation of Ilexsaponin A_1_

The leaves of *I. hainanensis* were collected from the Guangxi province, China, in October 2015. They were identified and authenticated by Ke Zan (National Institutes for Food and Drug Control, Beijing, China). A voucher specimen (no. 20151101) was deposited at the School of Traditional Chinese Medicine, Capital Medical University, China. Dried and pulverized leaves of *I. hainanensis* (5 kg) were extracted twice by the heat reflux method with six volumes of 75% ethanol for 1.5 h each. The solutions were filtered and evaporated with a rotary evaporator to yield 950 g extract. A fraction of the extract was loaded onto a D101 macroporous resin column and eluted with a gradient of EtOH/H_2_O (50:50 and 70:30, *v/v*). Saponins were mainly enriched in the 70% ethanol fraction as identified using reference substances. The 70% EtOH fraction was then subjected to Sephadex LH-20 column chromatography and eluted with MeOH. The subfractions containing saponins were combined and concentrated to obtain total saponins. IsA was purified from total saponins by *semi*-preparative HPLC (MeOH/H_2_O, 75:25, *v/v*). The IsA structure was determined by ^1^H-NMR, ^13^C-NMR, and MS. HPLC [Agilent HPLC-1200 Infinity, Santa Clara, CA, USA; Eclipse XDB-C_18_ column (4.6 × 250 mm, 5 μm; Agilent Technologies); UV detector 210 nm] revealed that the purity of IsA was higher than 95% in the area normalization method (Supplementary Figure S1).

### Animals

Male C57BL/6 mice (4–6 weeks old) obtained from the Vital River Laboratory Animal Technology (Beijing, China) were housed under standard conditions (12-h light–dark cycle, 60%–70% humidity, and 25 ± 2°C). After a 7-day adaptation period, mice were randomly assigned to four groups (*n* = 6): 1) Chow group, fed a standard chow diet (#D12450J, Research Diets, New Brunswick, NJ, USA); 2) HFD group, fed an HFD diet (containing 60 kcal% fat, #D12492, Research Diets, New Brunswick, NJ, USA); 3) IsA group (120 mg/kg), fed an HFD diet; and 4) FNB group (fenofibrate, 60 mg/kg, positive control, Beijing Yimin Pharmaceutical Company), fed an HFD diet. IsA, fenofibrate, and vehicle were administrated to the animals by oral gavage once a day for 8 weeks. All mice were housed with free access to chow/HFD and water. Body weight and food intake were recorded once a week for each animal. At the end of the experiment, mice were fasted for 12 h, and blood samples were collected by cardiac puncture under anesthesia before euthanasia. Livers, intestinal contents, intestinal tissues, and feces were collected and stored at −80°C.

### Dosage Information

The *I. hainanensis* preparation “Shanlvcha Jiangya tablets” has been used for treating hypertension and hyperlipidemia for about 40 years in clinics. The dosage of IsA in this study was selected based on our previous research and the human dosage of “Shanlvcha Jiangya tablets” (Zhao et al., 2019). The converted dosage of IsA from human to mice was about 120 mg/kg based on the Meeh–Rubner equation. The dosage of fenofibrate was used according to the clinical dosage and our preliminary experiments.

### Glucose Tolerance Test

For intraperitoneal glucose tolerance test (ipGTT), mice were fasted for 16 h prior to testing and intraperitoneally administered with 2.0 g/kg of glucose. Blood samples were drawn from the tail vein before injection or at 15, 30, 60, and 120 min after the injection, respectively. Glucose concentrations were measured with a OneTouch Ultra glucometer (Johnson, New Brunswick, NJ, USA), and the area under the curve (AUC) was calculated. Serum insulin levels were determined with an insulin ELISA kit (Merck-Millipore, Billerica, MA, USA). Insulin resistance (IR) was assessed using the index of homeostasis model assessment-IR (HOMA-IR): fasting blood glucose (mmol/L) × fasting blood insulin (mU/L)/22.5. All experimental procedures were carried out according to the instructions of the manufacturer.

### Biochemical Assays

Serum triglyceride (TG), total cholesterol (TC), alanine aminotransferase (ALT), aspartate aminotransferase (AST), and low-density lipoprotein cholesterol (LDL-c) levels were measured with commercial kits (Jiancheng Bioengineering Institute, Nanjing, China). Serum LPS was detected with a commercial ELISA kit (Cloud-Clone Corp., Houston, TX, USA). All assays were strictly performed according to the instructions of the manufacturer.

### Histopathological Analysis

A portion of the liver tissue was fixed with 4% paraformaldehyde for 12 h at 4°C, dehydrated, and embedded in paraffin. Sections (thickness of 4–5 µm) were stained with hematoxylin and eosin (H&E) and visualized under a Nikon Eclipse 80i optical microscope (Nikon, Tokyo, Japan). The NAFLD activity score (NAS) was used to provide a semiquantitative evaluation of three histological features, including steatosis (0–3), lobular inflammation (0–3), and hepatocellular ballooning (0–2).

Another portion of the liver tissue was placed in 30% sucrose solution for dehydration. The dehydrated liver sample was processed into frozen sections (thickness of 8 µm), stained with Oil red O (Servicebio G1016, Servicebio, Wuhan, China) for 10 min, and imaged by the Pannoramic digital slide scanner (Pannoramic Scan II, 3Dhistech Ltd., Budapest, Hungary).

### Quantification of Hepatic and Fecal Bile Acids

BA standards, including taurocholic acid (TCA), taurodeoxycholic acid (TDCA), and taurolithocholic acid (TLCA) were purchased from Sigma-Aldrich (St. Louis, MO, USA). Cholic acid (CA) and lithocholic acid (LCA) were purchased from Dalian Meilun Biotechnology Co., Ltd. (Dalian, China). Chenodeoxycholic acid (CDCA), deoxycholic acid (DCA), taurochenodeoxycholic acid (TCDCA), ursodesoxycholic acid (UDCA), and tauroursodesoxycholic acid (TUDCA) were purchased from Chengdu Herbpurify Co., Ltd. (Chengdu, China). Β muricholic acid (βMCA) and tauro-β muricholic acid (T-βMCA) were purchased from J&K Scientific., Ltd. (Beijing, China). Deuterated CA-2,2,4,4-d_4_ was purchased from C/D/N Isotopes, Inc. (Pointe-Claire, Quebec, Canada). Dehydrocholic acid (DHCA) was purchased from Shanghai Yuanye Bio-Technology Co., Ltd. (Shanghai, China).

For BA assessment, liver tissue and fecal samples were homogenized with 1 ml of ddH_2_O containing 200 ng of CA-d_4_ and 40 ng of DHCA, followed by centrifugation (4°C, 14,000 × *g*) for 10 min. The supernatant was loaded onto a preactivated Hypersep C18 solid phase extraction (SPE) column (Thermo Fisher Scientific, Shanghai, China). Then, 1 ml of ddH_2_O was added to the column to remove impurities, and 1 ml of methanol was used to elute the analytes. The methanolic solution was collected and evaporated to dryness under a stream of nitrogen gas at 37°C. The dried extract was reconstituted with 100-μl mobile phase from which 1 μl of the sample was injected into an Agilent 1,290 system (Agilent Technologies, USA) coupled with an AB Sciex 6500 QTRAP ^®^ mass spectrometer (Foster City, CA, USA) equipped with an ESI source. The LC-MS/MS method was referred to the method described previously (John et al., 2014).

### Bacterial DNA Extraction and 16S rRNA Gene Sequencing

Total microbial genomic DNA was extracted from cecal contents with the QIAamp DNA Stool Mini Kit (QIAGEN, Inc., Netherlands), following the instructions of the manufacturer. After DNA extraction, primers 341F (5′-ACT​CCT​ACG​GGA​GGC​AGC​AG-3′) and 806R (5′-GGACTACHVGGGTWTCTAAT-3′) were used to amplify the V3–V4 region of the bacterial 16S ribosomal RNA gene. Sequencing was performed on an Illumina MiSeq PE300 platform (BGI Shenzhen Co., Ltd., Guangdong, China).

### Bile Salt Hydrolase Activity Analysis

Fecal total protein was prepared from 50 mg of fecal samples in 250 μl of PBS (pH 7.4) by sonication. Protein concentrations were determined with a BCA protein assay kit (Applygen, Beijing, China), and samples were diluted to 2 mg/ml with PBS as the protein working solution. Bacterial BSH activity was measured based on CA generation from TCA in the feces. Briefly, incubation was carried out with 3 mM sodium acetate buffer (pH 5.2) containing 0.1 mg/ml of fecal protein and 0.1 mM of D_4_-TCA in a volume of 200 µl. After a 20-min incubation at 37°C, reactions were stopped by plunging the samples into dry ice. Next, 100 µl of methanol was added per reaction mix, followed by centrifugation (12,000 × *g*, 20 min). Finally, 1 µl of the supernatant was analyzed by the method in the *Quantification of hepatic and fecal bile acids* section.

### Quantitative Real-Time PCR

Total RNA from the liver and ileal tissue samples (20 mg) was extracted with the RNA prep pure tissue kit (Tiangen Biotech Co., Ltd., Beijing, China) according to the instructions of the manufacturer. The concentrations of total RNA were measured using the Nanodrop 2000C spectrophotometer (Thermo Fisher Scientific, Waltham, MA, USA). Then, 1 μg of total RNA was reverse transcribed with random hexamer primers to form the cDNA template with the FastQuant RT Kit (Tiangen Biotech Co., Ltd., Beijing, China). The quantitative real-time PCR reaction mixture was set up with SuperReal PreMix Plus (Tiangen Biotech Co., Ltd., Beijing, China). An ABI QuantStudio^®^ 5 Real-Time PCR System (Thermo Fisher Scientific, Hudson, NH, USA) was used for relative quantification of interleukin-6 (IL-6), tumor necrosis factor-α (TNF-α), and nuclear factor kappa-B (NF-κB) mRNAs with the SYBR green probe. A Bio-Rad MiniOption™ Real-Time PCR Detection System (Bio-Rad, Hercules, CA, USA) was used for relative quantification of CYP7A1, CYP7B1, CYP27A1, FXR, small heterodimer partner (SHP), NTCP, BESP, and FGF15 mRNAs (primers listed in the Supplementary Material). Gene expression was normalized to the levels of β-actin. Relative gene expression was calculated by the 2^−△△Ct^ method.

### Western Blotting

Total protein was isolated from ileum samples, and protein concentrations were measured with the BCA protein assay kit (Applygen, Beijing, China). A 6-μg/μl protein extract was mixed with loading buffer and denatured by boiling at 95°C for 5 min. The denatured proteins (48 μg) were resolved by 8% SDS-PAGE and transferred to polyvinylidene difluoride (PVDF) membranes (0.45 μm, Merck Millipore, USA). The membranes were blocked with 5% BSA-TBST (Servicebio Technology, Wuhan, China) at room temperature for 1 h, incubated with primary antibodies against FXR (1:1,000; Cell Signaling Technology, #72105) overnight at 4°C, and further incubated with horseradish peroxidase-conjugated secondary antibodies. The PVDF membranes were stripped and reprobed for β-tubulin as a loading control with anti-β-tubulin antibodies (1:2,000; Proteintech Group, Rosemont, USA, #10094-1-AP). Immunoreactive bands were developed using the Super ECL Plus kit (Applygen, China) and visualized with the Fusion FX6 XT System (Vilber, France). The ImageJ software was used for quantitation.

### Statistical Analysis

Statistical analyses were performed with the statistical computer package GraphPad Prism version 8 (GraphPad Software Inc., San Diego, CA, USA). Data were expressed as mean ± SEM. Statistical comparisons were made by one-way ANOVA with *post-hoc* Tukey’s test. For intestinal microbial composition analysis, data were compared by the Kruskal–Wallis test. Differences were considered to be significant at *p* < 0.05.

## Results

### Ilexsaponin A_1_ Reduces Body Weight Gain and Improves Insulin Resistance in Nonalcoholic Fatty Liver Disease Mice

**FIGURE 1:**
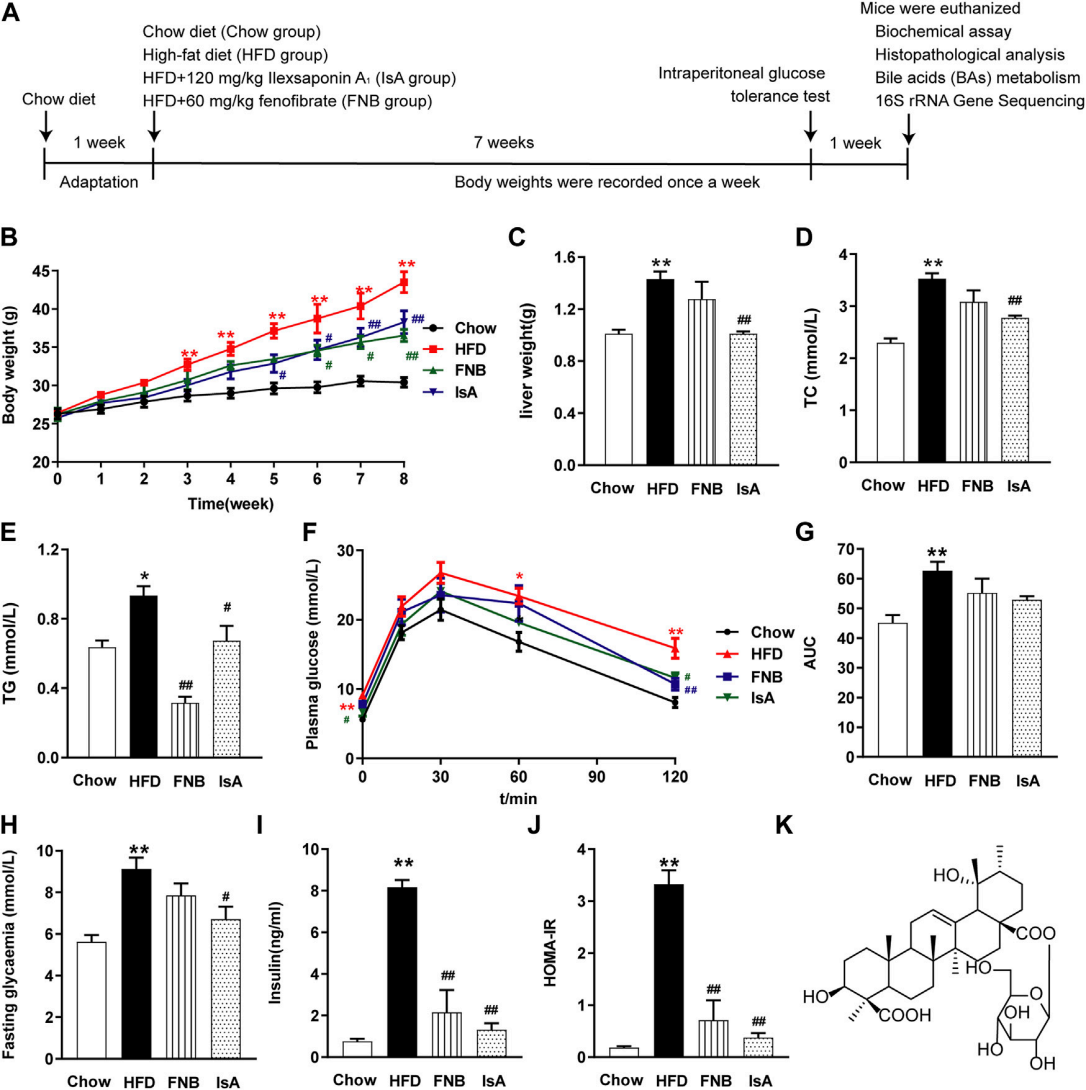
Ilexsaponin A_1_ (IsA) attenuates body weight gain and insulin resistance (IR) in a high-fat diet (HFD)-induced nonalcoholic fatty liver disease (NAFLD) mouse model. **(A)** C57BL/6 mice were conditioned for 1 week. For the subsequent 8 weeks, mice in diverse groups were, respectively, administrated normal diet, HFD, and HFD with IsA or fenofibrate. Body weights in each group were determined weekly. At the endpoint, mice were euthanized, and associated indications were investigated. **(B)** Body weights during the modeling time. **(C)** Liver weights. **(D)** Serum total cholesterol (TC) levels. **(E)** Serum triglyceride (TG) levels. **(F)** Intraperitoneal glucose tolerance test (ipGTT) results. **(G)** Areas under the curves (AUCs) of ipGTT. **(H)** Fasting glycemia. **(I)** Serum insulin levels. **(J)** Homeostasis model assessment-insulin resistance (HOMA-IR) index. **(K)** Structure of IsA. Data are mean ± SEM (*n* = 6). ^*^ *p* < 0.05, ^**^ *p* < 0.01 *vs*. chow group; ^#^ *p* < 0.05, ^##^ *p* < 0.01 *vs*. HFD group.

Mice were fed an HFD for 8 weeks, and IsA was supplemented at the beginning of the time period and daily thereafter (Figure 1A). With HFD administration, the body and liver weights of mice were significantly increased compared with the chow group. The liver and body weight gains were attenuated in the IsA group compared with the HFD group (Figures 1B, C). It is noteworthy that compared with the HFD group, serum TC levels were significantly decreased in the IsA group, with lower values than that of the mice treated with fenofibrate (Figure 1D). In the fenofibrate group, body weights and serum TG levels were significantly reduced compared with the HFD group (Figures 1B, E).

To evaluate insulin sensitivity in mice, we performed ipGTT, tested fasting blood glucose and insulin, and calculated the HOMA-IR index (Figures 1F–J). After 8 weeks of HFD, the ipGTT-AUC (Figures 1F, G), fasting blood glucose concentration (Figure 1H), insulin concentration (Figure 1I), and HOMA-IR index (Figure 1J) were enhanced remarkably in the HFD group compared with the chow group. In the IsA group, fasting blood glucose concentration, fasting insulin concentration, and HOMA-IR index were decreased significantly compared with the HFD group. Fenofibrate treatment significantly decreased serum insulin levels and the HOMA-IR index. These results indicated that IsA supplementation alleviated IR in NAFLD mice.

### Ilexsaponin A_1_ Alleviates Liver Steatosis and Inflammation in Nonalcoholic Fatty Liver Disease Mice

**FIGURE 2:**
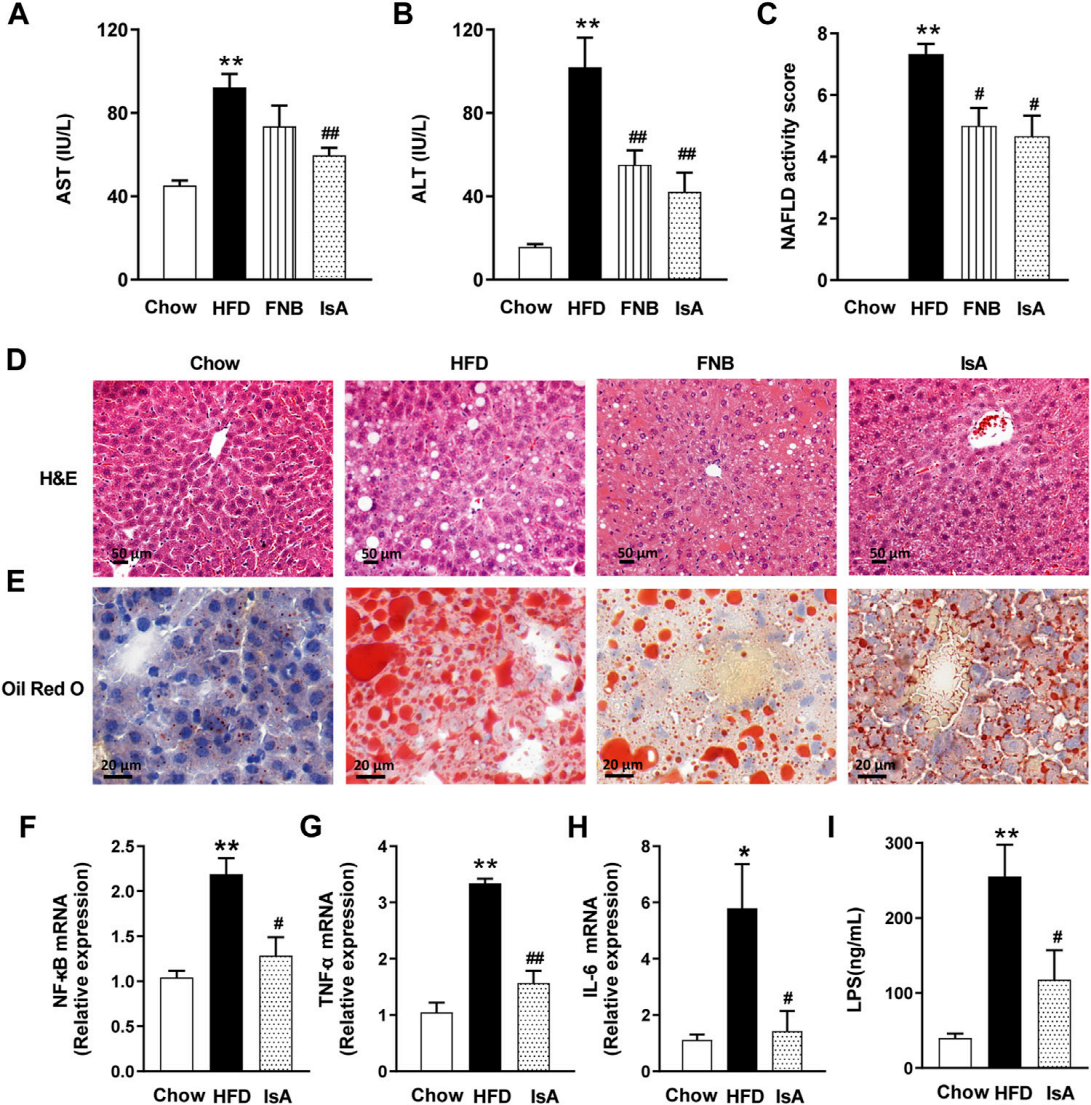
IsA alleviates liver steatosis and inflammation. **(A)** Serum aspartate aminotransferase (AST) levels. **(B)** Serum aminotransferase (ALT) levels. **(C)** NAFLD activity scores. **(D)** Hematoxylin and eosin (H&E) liver sections (scale bar: 50 μm). **(E)** Oil-red O liver sections (scale bar: 20 μm). **(F–H)** Relative mRNA expression levels of nuclear factor kappa-B (NF-κB), tumor necrosis factor-α (TNF-α), and interleukin-6 (IL-6) in hepatic tissue samples assessed by qRT-PCR. **(I)** Serum LPS levels determined by ELISA. Data are mean ± SEM (*n* = 6). ^*^ *p* < 0.05, ^**^ *p* < 0.01 *vs.* chow group; ^#^ *p* < 0.05, ^##^ *p* < 0.01 *vs*. HFD group.

Serum ALT and AST levels were significantly increased in HFD-fed mice compared with the chow group. IsA supplementation significantly reduced serum ALT and AST levels in mice. Serum AST levels were significantly decreased in the fenofibrate group (Figures 2A, B). Compared with the chow mice, mice fed with HFD showed severe changes in liver morphology (H&E and Oil red O staining), including increased number and volume of lipid droplets. IsA and fenofibrate treatments, respectively, resulted in reduced number and size of lipid droplets in liver cells, and IsA showed better treatment effects than the positive control (Figures 2D, E).

We evaluated the inflammatory response in the hepatic tissue by the qRT-PCR method. HFD feeding increased the mRNA expression levels of NF-κB, which were reduced by IsA supplementation (Figure 2F). The mRNA expression levels of TNF-α and IL-6, two inflammatory genes regulated by NF-κB, were significantly increased in the HFD group, which were reduced in the IsA group (Figures 2G, H). These results indicated that IsA treatment suppressed HFD-induced proinflammatory response.

### Ilexsaponin A_1_ Reduces the Accumulation of Hepatic Bile Acids in Nonalcoholic Fatty Liver Disease Mice

**FIGURE 3:**
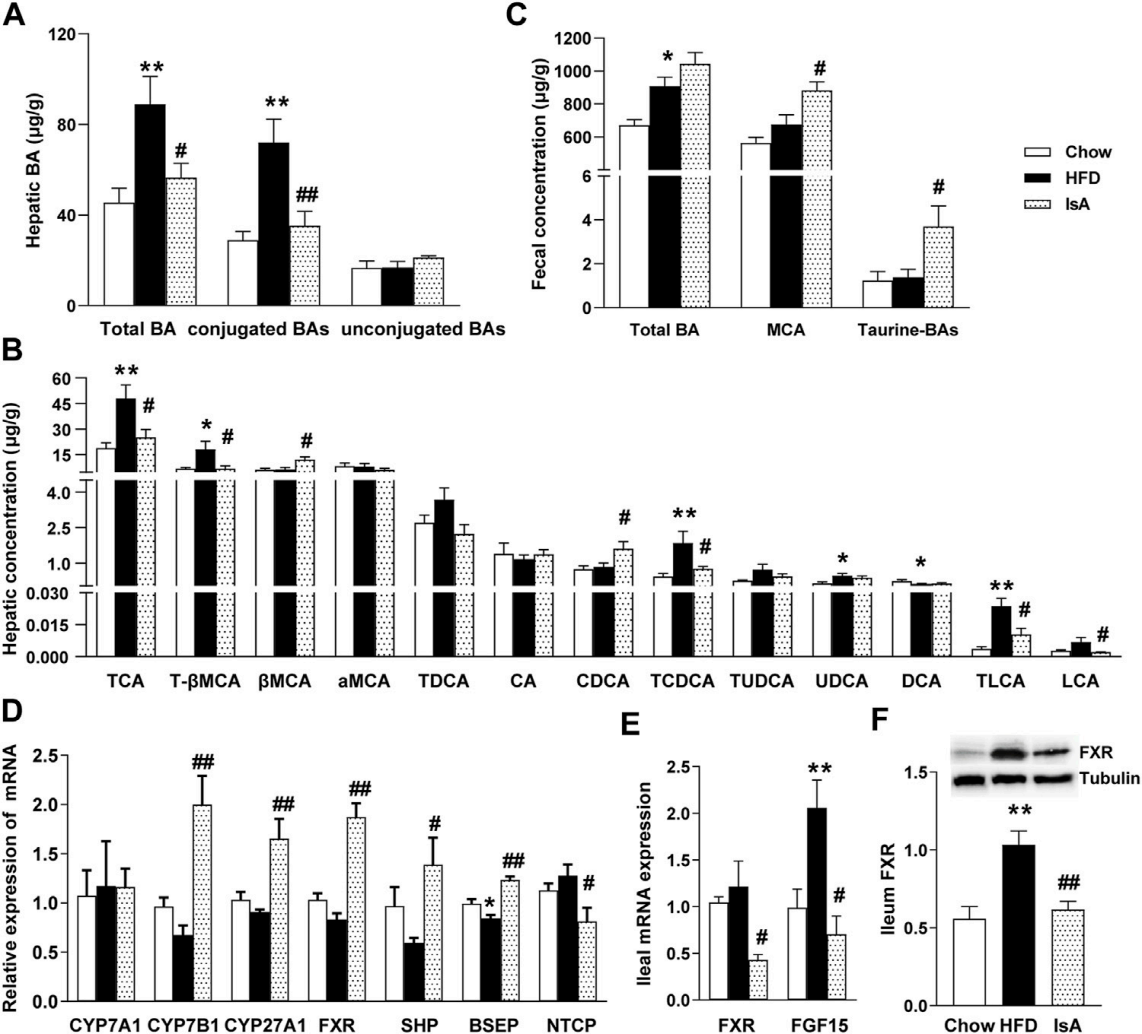
Effects of IsA on bile acid (BA) metabolism in mice. **(A)** Concentrations of hepatic total BAs, conjugated BAs, and unconjugated BAs. **(B)** Concentrations of hepatic BAs. **(C)** Concentrations of fecal total BAs, MCA, and taurine-BAs. **(D)** Relative mRNA expression levels of hepatic cholesterol 7α-hydroxylase (CYP7A1), sterol 27-hydroxylase (CYP27A1), cholesterol 7b-hydroxylase (CYP7B1), farnesoid X receptor (FXR), small heterodimer partner (SHP), bile salt export pump (BSEP), and Na^+^-taurocholic acid cotransporting polypeptide (NTCP). **(E)** Relative mRNA expression levels of ileal FXR and fibroblast growth factor 15 (FGF15). **(F)** FXR protein levels assessed by Western blot. Data are mean ± SEM (*n* = 5–6). ^*^ *p* < 0.05, ^**^ *p* < 0.01 *vs.* chow group; ^#^ *p* < 0.05, ^##^ *p* < 0.01 *vs*. HFD group.

In order to investigate the effect of IsA on endogenous BAs, targeted metabolomics analysis of BAs was performed in the mouse liver and fecal samples. Compared with chow-fed mice, HFD-fed animals displayed significantly increased total hepatic BA levels. IsA treatment alleviated this effect significantly (Figure 3A). Statistical analyses showed that the hepatic levels of conjugated BAs increased in HFD-fed mice, but decreased in IsA-treated mice (Figure 3B). Notably, hepatic CDCA levels were significantly increased, while T-βMCA concentrations were significantly decreased in the livers of IsA-treated mice (Figure 3B). It is known that CDCA is an endogenous FXR agonist, and T-βMCA is an FXR antagonist. Thus, IsA supplementation was likely to increase hepatic FXR activity.

The IsA group mice showed increased fecal levels of MCA, which are the main BAs excreted in mouse feces. The fecal levels of taurine-conjugated BAs were significantly increased, implying enhanced FXR antagonism in the intestine (Figure 3C).

### Ilexsaponin A_1_ Alters the Expression of Genes Involved in Bile Acid Metabolism in Nonalcoholic Fatty Liver Disease Mice

To investigate the modulatory mechanism of BAs induced by IsA treatment, the mRNA expression levels of CYP7A1, CYP7B1, CYP27A1, FXR, SHP, BSEP, and NTCP in the liver, and FXR and FGF15 in the ileum were determined, as genes involved in BA generation, metabolism, and transportation. The mRNA expression levels of hepatic BA synthetic genes, CYP7B1 and CYP27A1, were significantly increased in the IsA treatment group compared with the HFD group (Figures 3D). The relative mRNA expression levels of ileal FXR and FGF15 in the IsA group were significantly lower than those of the HFD group. Ileal FXR protein expression was reduced in the IsA group compared with the HFD group (Figures 3E, F). The increased gene expression in the alternative BA synthetic pathway led to increased bioconversion from cholesterol to BAs and enhanced CDCA production rather than CA synthesis. Compared with the HFD group, IsA-treated mice showed increased mRNA expression levels of hepatic FXR and SHP. The gene expression levels of the BA excretory transporter BSEP were significantly increased, and the basolateral uptake transporter NTCP was significantly downregulated in IsA-treated mice (Figure 3D)*.* The above findings revealed that IsA promoted BA efflux from the liver into bile *via* an induction of FXR-target gene BSEP expression, and reduced hepatic uptake through NTCP downregulation*.*

### Ilexsaponin A_1_ Increases the Proportion of Bile Salt Hydrolase-Producing Bacteria and Bile Salt Hydrolase Activity in Nonalcoholic Fatty Liver Disease Mice

**FIGURE 4:**
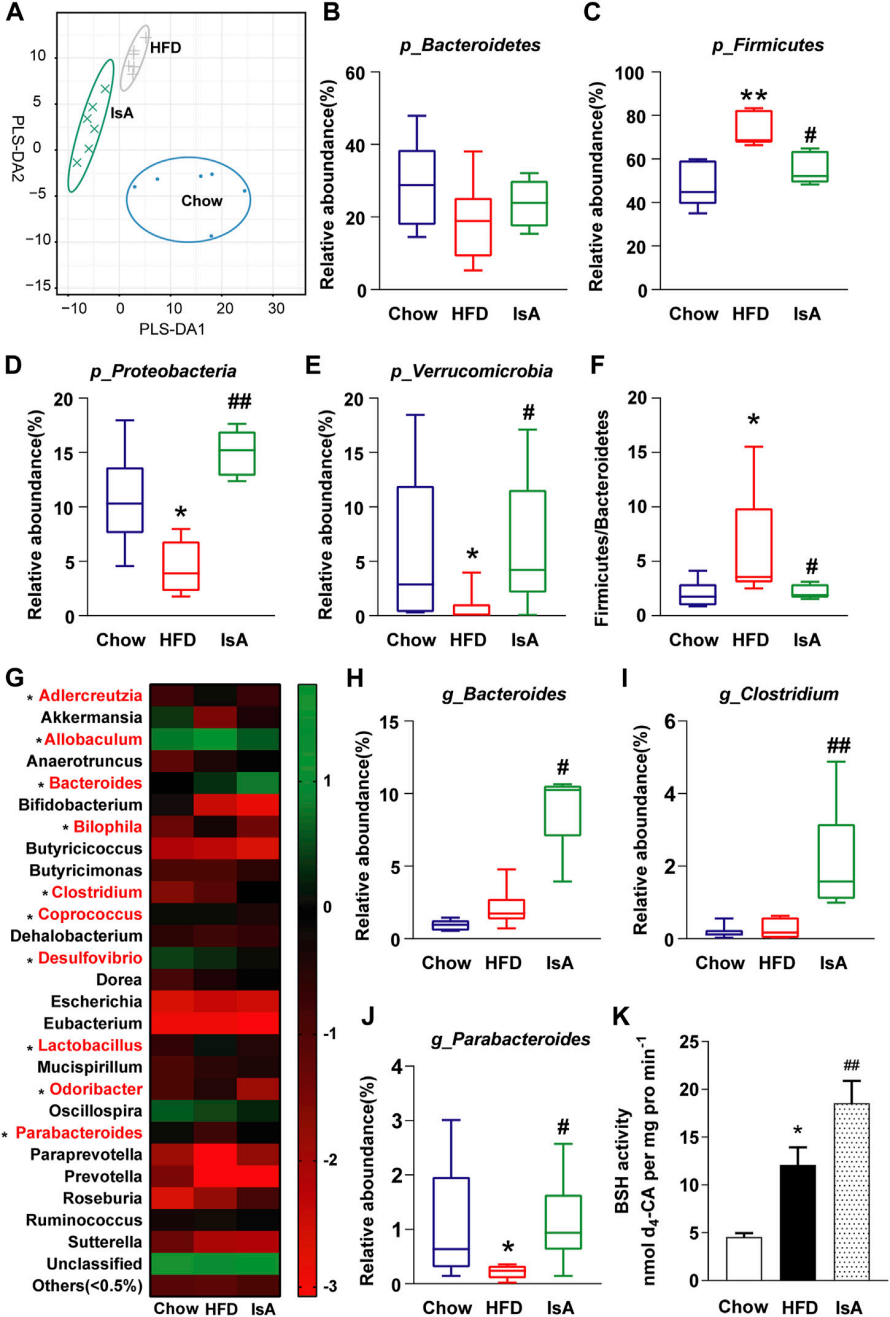
Effects of IsA on the gut microbiota and bile salt hydrolase (BSH) activity in mice. **(A)** Partial least squares discrimination analysis (PLS-DA) at the phylum level in each group. The horizontal and vertical axes indicate the top two components. **(B**–**E)** The relative abundance levels of Bacteroidetes, Firmicutes, Proteobacteria, and Verrucomicrobia. **(F)** Firmicutes/Bacteroidetes ratios. **(G)** Heat map of the gut microbiota in response to IsA at the genus level. The color red shows the 10 genera significantly changed by IsA. **(H**–**J)** Relative abundance levels of the *Bacteroides*, *Clostridium*, and *Parabacteroides* genera. Statistical analysis was performed by the Kruskal–Wallis test (*n* = 6). ^*^ *p* < 0.05, ^**^ *p* < 0.01 *vs*. chow group; ^#^ *p* < 0.05, ^##^ *p* < 0.01 *vs*. HFD group. **(K)** BSH activities (*n* = 5). ^*^ *p* < 0.05, ^**^ *p* < 0.01 *vs*. chow group; ^#^ *p* < 0.05, ^##^ *p* < 0.01 *vs*. HFD group.

The gut microbiota analysis revealed that HFD and IsA drastically altered microbial composition compared with the chow group, according to partial least squares discrimination analysis (PLS-DA) (Figure 4A). The relative abundance levels of operational taxonomic units (OTUs) (%) in Firmicutes were significantly decreased in the IsA group, while Verrucomicrobia and Proteobacteria were increased compared with the HFD group at the phylum level (Figures 4B–E). The ratios of Firmicutes/Bacteroidetes were significantly increased in the HFD group, and reduced in the IsA group, as shown in Figure 4F. The heat map (Figure 4G) shows the bacterial genera with the most substantial changes in abundance after exposure to IsA. Of the 10 genera significantly changed by IsA (bacteria in red in Figure 4G), five were involved in BA metabolism, including *Bacteroides*, *Bilophila*, *Clostridium*, *Lactobacillus*, and *Parabacteroides* (Gerard, 2013). The abundance levels of BSH-enriched genera, such as *Bacteroides*, *Clostridium*, and *Parabacteroides*, were significantly increased in the IsA group (Figures 4H–J) (Wahlstrom et al., 2016; Ridlon et al., 2020). BSH activity assessment in mouse fecal samples showed that BSH activity in the IsA group was also increased (Figure 4K). These data showed that IsA treatment increased the relative abundance of BSH-producing bacteria and enhanced BSH activity, leading to increased BA deconjugation and excretion in the ileum.

## Discussion

Accumulating evidence suggests that BA metabolism is associated with liver disease and may be a potential target for its treatment (Jung et al., 2021). Many natural products have been shown to alleviate or prevent NAFLD by regulating BA metabolism (Huang et al., 2019; Xiong et al., 2021). In the present study, IsA effectively ameliorated hepatic steatosis and related metabolic dysfunctions in HFD-induced NAFLD mice. In addition, mechanistic insights into the regulatory effects of IsA on the gut microbiota, as well as the production and excretion of BAs to increase cholesterol consumption are discussed.

HFD is widely used to induce NAFLD in mice. In this study, hepatic lipid homeostasis was disrupted by HFD, and fat accumulation was observed in the HFD group. Administration of IsA for 8 weeks significantly reduced body weight gain and hepatic steatosis, decreased serum ALT and AST levels, and improved IR compared with untreated HFD mice.

NAFLD is involved in chronic and low-grade inflammation (Luo and Lin, 2020). This study showed that IsA treatment improved liver injury and inflammation. Increased hepatic BAs could cause cytotoxicity and stimulate the secretion of proinflammatory cytokines (Li et al., 2017). Under these conditions, both cholestasis and inflammation are intensified, which can promote the development of NAFLD (Zhang and Wang, 2011; Li et al., 2013). In this study, IsA administration reduced hepatic toxic BAs (LCA, TLCA, and TCDCA) in mice (Woolbright et al., 2014; Amonyingcharoen et al., 2015; Xie et al., 2016). IsA also activated hepatic FXR, which may regulate inflammation by inhibiting the activation of the proinflammatory transcription factor NF-κB (Wang et al., 2008; Lefebvre et al., 2009). The reduced NF-κB mRNA levels and hepatic inflammatory cytokines (TNF-α and IL-6) confirmed the above notion, resulting in reduced hepatic inflammation in HFD-induced liver injury.

IsA was found to reduce serum TC levels more significantly than fenofibrate. A previous study showed that BA biosynthesis and excretion play a vital role in maintaining cholesterol homeostasis (Huang et al., 2019). BA homeostasis is regulated by the FXR–FGF15 axis (Jia et al., 2020). Inhibition of intestinal FXR induces BA synthesis *via* the downregulation of ileal FGF15 (Huang et al., 2019). IsA-treated mice showed downregulated ileum FXR and FGF15 mRNA expression levels. Hepatic gene levels of enzymes related to BA synthesis suggested the alternative pathway of hepatic BA synthesis was promoted, with increased mRNA levels of CYP7B1 and CYP27A1, and enhanced hepatic CDCA levels in the IsA group. The increased hepatic CDCA production could promote hepatic FXR–SHP signaling and inhibit BA synthetic enzymes, mainly in the classical pathway (Jia et al., 2020). Ultimately, the combined regulation of intestinal FXR–FGF15 and hepatic FXR–SHP in hepatic BA synthesis results in increased expression of CYP7B1 in the alternative pathway, and the expression of CYP7A1 remained unchanged in the IsA group. These data suggested that IsA promoted BA synthesis from the alternative pathway, leading to increased consumption of cholesterol.

In this study, the enhanced levels of BA synthetic enzymes resulted in the accumulation of Bas in the liver. Reducing hepatic Bas could be achieved by multiple strategies such as increasing BA flow and inhibiting BA reabsorption from the intestine (Pathak et al., 2017). BSEP is a major transporter involved in the secretion of Bas from hepatocytes into bile, and this process is tightly regulated at the transcriptional and posttranscriptional levels by several liver-enriched transcription factors, for example, FXR (Ren et al., 2021). NTCP is a transporter that uptakes bile acids from portal blood into hepatocytes (Appelman et al., 2021). This study showed that IsA significantly increased hepatic CDCA levels and decreased T-βMCA amounts, thereby elevating hepatic FXR mRNA expression. IsA significantly decreased hepatic uptake and increased the efflux of BAs through decreased expression of the hepatic uptake transporter NTCP and upregulated efflux transporter BSEP. This would reasonably be expected to result in a decrease in total hepatic BAs by accelerating the efflux of hepatic BAs and inhibiting their uptake.

Given that IsA accelerated BA production and efflux in the liver, its effect on fecal excretion was also investigated. Fecal BA excretion is closely related to the gut microbiota. Gut bacteria-derived BSH is a major enzyme that catalyzes the “gateway” reaction in the bacterial metabolism of conjugated BAs to produce deconjugated BAs (Begley et al., 2006). Strains of probiotics with BSH activity are considered to be capable of reducing body weight gain and serum cholesterol levels (Joyce et al., 2014). In this study, IsA treatment regulated the microbial flora with an enrichment of BSH-containing genera, including *Bacteroides* and *Clostridium*, and increased BSH activity. The deconjugation of BAs was increased in fecal excretion, especially that of MCA (accounting for about 85% of fecal total BAs) because deconjugated BAs are less hydrophilic and less likely to be reabsorbed (Degirolamo et al., 2014). What is more, although the HFD group showed an increase in BA deconjugation and higher BA fecal loss like the IsA group, the BA synthetic enzymes and transporters were regulated the opposite way, which might explain why the same effect were not observed on the HFD group. Thus, IsA increased BA synthesis, decreased cholesterol levels, and increased the excretion of fecal BAs (mainly MCA).

## Conclusion

**FIGURE 5:**
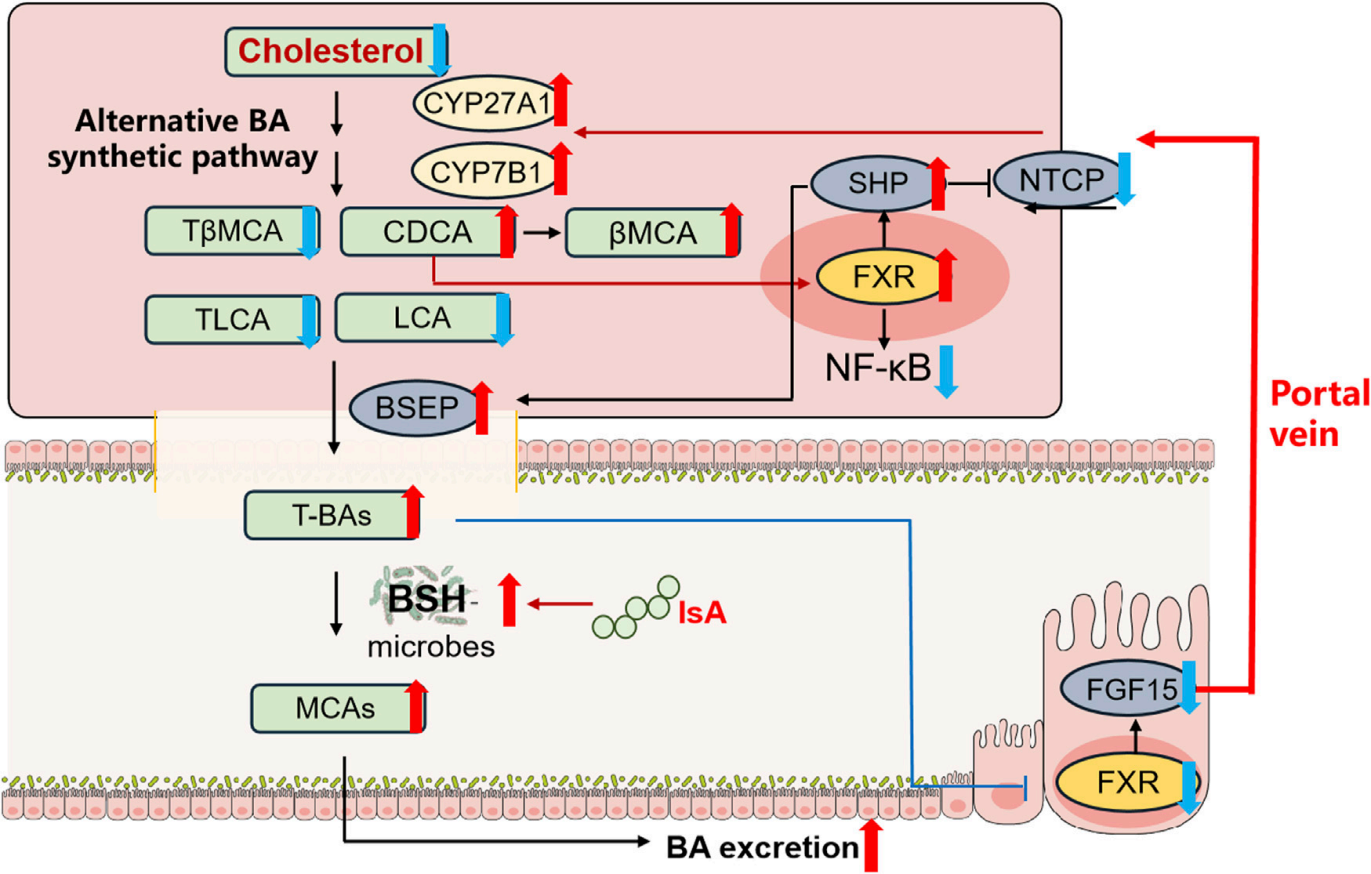
IsA protects against HFD-induced NAFLD in mice by regulating BA metabolism. Red and blue arrows indicate upregulation and downregulation after IsA treatment, respectively. This effect potentially involves (1) induced hepatic alternative BA synthetic pathway to increase cholesterol consumption, (2) accelerated BA efflux and suppressed BA uptake in the liver by regulating BSEP and NTCP, and (3) increased BSH-containing gut bacteria to increase fecal BA excretion.

In summary, we report that IsA treatment modulates BA metabolism in NAFLD mice induced by HFD for the first time. IsA significantly attenuated serum TC, liver steatosis, and inflammation with reduced hepatic BAs in HFD-induced NAFLD mice. These changes potentially involve 1) inhibited intestinal FXR signaling and promoted hepatic BA synthesis in the alternative pathway to increase cholesterol consumption, 2) accelerated BA efflux and suppressed BA uptake in the liver by regulating BSEP and NTCP, and 3) increased BSH-containing gut bacteria, which may contribute to increased fecal MCA excretion (Figure 5A). Taken together, these results suggest that IsA may be used to prevent NAFLD associated with hypercholesterolemia.

## Data Availability

The datasets presented in this study can be found in online repositories. The names of the repository/repositories and accession number(s) can be found below: NCBI (accession: PRJNA776658).
