# Supplementary material for: Ilexsaponin A1 Ameliorates Diet-Induced Nonalcoholic Fatty Liver Disease by Regulating Bile Acid Metabolism in Mice
Source: Front Pharmacol. 2021 Dec 14;12:771976. doi: 10.3389/fphar.2021.771976 (PMC8712733; doi:10.3389/fphar.2021.771976)
Supplement: Supplementary file 1 [file DataSheet1.docx]

Supplementary Material

Ilexsaponin A_1_ Ameliorates Diet-Induced Non-alcoholic Fatty Liver Disease by Regulating Bile Acid Metabolism in Mice

Wen-wen Zhao^1,2^, Meng Xiao^1,3^, Xia Wu^1^, Xiu-wei Li^1^, Xiao-xi Li^1^, Ting Zhao^1^, Lan Yu^1^ and Xiao-qing Chen^1*^

^1^School of Traditional Chinese Medicine, Capital Medical University, Beijing 100069, China

^2^Department of Pharmacy, Beijing Children's Hospital, Capital Medical University, National Center for Children's Health, Beijing 100045, China

^3^National Institutes for Food and Drug Control, Beijing 102629, China

*** Correspondence:**Dr. Xiaoqing Chen
cxqcpu@163.com

Keywords: Ilexsaponin A_1_, non-alcoholic fatty liver disease, bile acid, gut microbiota, farnesoid X receptor

**1 Figure S1**


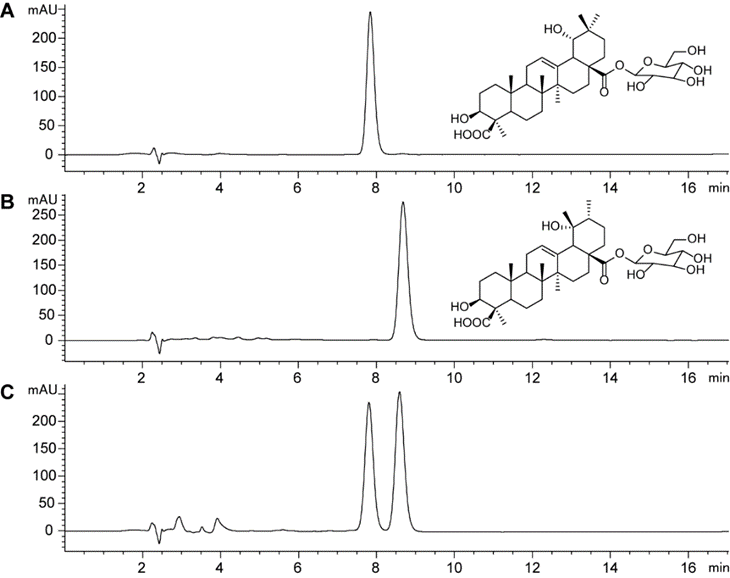


Figure S1 | The HPLC chromatogram of Ilexsaponin A_1_

**2 The PCR primers**

The PCR primers were as follows (5′→3′, forward and reverse): IL-6, 5′- CCG GAG AGG AGA CTT CAC -3′ and 5′- TCC ACG ATT TCC CAG AGA -3′; TNF-α, 5′- TCA GTT CCA TGG CCC AGA C -3′ and 5′- GTT GTC TTT GAG ATC CAT GCC ATT -3′; NF-κB, 5′- TCA GGA AGA GGT TTG GAT GC -3′ and 5′- AGC CCC TAA TAC ACG CCT CT -3′; CYP7A1, 5′- CCT CTG GGC ATC TCA AGC AA -3′ and 5′- AAT GGC ATT CCC TCC AGA GC -3′; CYP7B1, 5′- AGT TGC AGC GTC TCT TCC AT -3′ and 5′- CCT TGC TCC CTC AGA AAC TG -3′; CYP27A1, 5′- GAG TAC GGA GGG TCC AGG AA -3′ and 5′- GTC CCA AAG GAG GTT GTC CA -3′; FXR, 5′- TGG GTA CCA GGG AGA GAC TG -3′ and 5′- GTG AGC GCG TTG TAG TGG TA -3′; SHP, 5′- AGC TGG GTC CCA AGG AGT AT -3′ and 5′- TGA TAG GGC GGA AGA AGA GA -3′; BSEP, 5′- GGA CAA TGA TGT GCT TGT GG -3′ and 5′- CAC ACA AAG CCC CTA CCA GT-3 -3′; NTCP, 5′- ACT GGC TTC CTG ATG GGC TAC -3′ and 5′- GAG TTG GAC GTT TTG GAA TCC T -3′; FGF15, 5′- AAA ACG AAC GAA ATT TGT TGG AA -3′ and 5′- ACG TCC TTG ATG GCA ATC G -3′; β-actin, 5′- CAT CCG TAA AGA CCT CTA TGC CAA C-3′ and 5′- ATG GAG CCA CCG ATC CAC A -3′.
